# Dataset on the exploratory factor structure of organizational health performance for micro and small enterprises: The initial research stage

**DOI:** 10.1016/j.dib.2025.112317

**Published:** 2025-11-21

**Authors:** Uun Sunarsih, Hendryadi, Irsan Tricahyadinata, Syahrul Effendi, Faris Faruqi, Harry Budiantoro, Santi Retno Sari

**Affiliations:** a Sekolah Tinggi Ilmu Ekonomi Indonesia Jakarta, Jakarta, Indonesia; b Economics and Business Faculty, Mulawarman University, Samarinda, Indonesia; c Business Creation Program, Management Department, BINUS Business School Undergraduate Program, Bina Nusantara University, Jakarta, Indonesia; d Economics and Business Faculty, YARSI University, Jakarta, Indonesia; e Economics and Business Faculty, National University, Jakarta, Indonesia

**Keywords:** Organizational health performance data, Micro and small enterprises, Exploratory factor analysis, Indonesia

## Abstract

This dataset was collected during the initial research phase to investigate the factor structure of organizational health performance in micro and small enterprises (MSEs). A time-lag cross-sectional survey was conducted to collect data from 428 pairs of owner-employee responses in two provinces: Jakarta and West Java, Indonesia. Exploratory Factor Analysis (EFA) was conducted to identify the underlying factor structure. Of 35 items, 32 were grouped into six factors: institutional resources, leader involvement, work environment quality, organizational values, operational stability, and economic performance. For researchers, this dataset provides a validated, multi-dimensional instrument to measure organizational health in the MSE context. Moreover, this dataset also provides practitioners with a comprehensive tool for assessing the strengths and weaknesses of MSEs, identifying areas for intervention, which are crucial drivers of economic development.

Specifications Table

**Table:** 

Subject	Social Sciences
Specific subject area	*Micro and small enterprises, organizational health.*
Type of data	Table Processed, .XLS.
Data collection	A total of 428 paired matched sets of owner-employee ratings completed this survey, who were selected using a convenience sampling method at two different times. The sampling was done in the Jakarta and West Java provinces, Indonesia. The data were evaluated using the Exploratory Factor Analysis (EFA)*.*
Data source location	• Jakarta Province, Indonesia • West Java Province, Indonesia
Data accessibility	Repository name: Mendeley Data Data identification number:10.17632/84n5psm3hb.1 Direct URL to data: https://data.mendeley.com/datasets/84n5psm3hb/1 Data Codebook: http://repository.stei.ac.id/16066/
Related research article	*No* research paper refers to the dataset*.*

## 1 Value of the Data

• The dataset provides a foundational framework for assessing organizational health in Indonesia's MSE sector.
• The dataset provides an empirically tested and reliable tool (comprising 32 items across six factors) to quantify the previously less-defined construct of organizational health performance in the specific context of Micro and Small Enterprises.
• The data was collected from 428 paired responses of owners and their employees, providing a multi-faceted and less biased view of internal organizational dynamics, which is a valuable asset for in-depth research on MSEs.
• The dataset is reusable for other researchers to conduct follow-up studies, such as Confirmatory Factor Analysis (CFA), comparative studies across different regions or industries, and to investigate the relationship between organizational health and other outcome variables.
• This dataset is also essential for identifying weaknesses and facilitating targeted interventions for policymakers and related stakeholders to enhance organizational health in the MSE context.
• This dataset is an excellent resource for statistics and methodology courses. Students can use it to gain hands-on experience with a wide range of techniques, including understanding scale development and factor extraction; calculating Cronbach's alpha to assess the internal consistency of the six identified factors; analyzing differences between owner and employee responses; and exploring relationships between the six factors of organizational health and a potential outcome like economic performance..

## 2 Background

Organizational health (OH) refers to an organization's overall condition and effectiveness in achieving its goals while ensuring sustainability. This concept has gained significant attention due to its close relationship with functionality, organizational vitality [1], and the ability to operate efficiently, adapt to changes, and meet objectives [2]. However, while extensive research on OH has focused on larger sectors [[3], [4], [5], [6]], the relevance of organizational health tools in micro and small enterprises (MSEs) remains underexplored. Unlike larger organizations, MSEs often rely on short-term strategies and operate with constrained financial resources, a limited workforce, and minimal technological capabilities, which makes them fundamentally different [7]. Consequently, existing OH measures may not fully capture the unique dynamics and vulnerabilities of MSEs; therefore, adjustments are needed. Hence, this dataset focuses on the initial development of organizational health scales tailored specifically for MSEs in developing countries.

This data represents the initial test of an organizational health instrument designed for the micro and small enterprise (MSE) sector. The research process started with qualitative Focus Group Discussions (FGDs) involving eight management academics, two government policymakers, and four practitioners. This phase helped define the key dimensions relevant to micro and small enterprises (MSEs): institutional resources, leader involvement, work environment quality, organizational values, operational stability, and economic performance. The next step involved collecting data through a time-lag cross-sectional survey from 428 owner-employee pairs in Indonesia. This dataset underwent Exploratory Factor Analysis (EFA) to test whether the six identified dimensions would emerge from MSE responses.

## 3 Data Description

The data were derived from 35 questionnaire items designed to measure organizational health in the MSE sector. The data questionnaire is available as a supplementary file on Mendeley (see https://data.mendeley.com/datasets/84n5psm3hb/1), and the codebook was available in the repository: http://repository.stei.ac.id/16066

The draft questionnaire initially consisted of 35 items, developed based on focus group discussions (FGD) involving eight academics (with doctoral degrees in strategic management, marketing, accounting, and finance), two government representatives specializing in regional micro and small business development, and four practitioners. Following validation by a panel of experts, their recommendations revised seven items (i.e., OH1, OH2, OH24, OH27, OH33, OH34, and OH35). The FGD identified six key factors for measuring organizational health in the micro and small enterprise (MSE) sector: institutional resources, leader involvement, work environment quality, organizational values, operational stability, and economic performance.

Different point scales were used for the questionnaire items to ensure contextual appropriateness, as recommended by expert panels. For institutional resources, MSE's owners were asked to provide answers on a 3-point scale, ranging from 1 (No) to 2 (in process) to 3 (Yes). 3-point scales are used because of their simplicity and can improve the clarity and usability of responses without compromising validity and reliability [7]. For example, the items ``Does your business have a Business Identification Number (NIB)?'' and ``Have you received any government funding assistance to support your business?'' do not require a complex scale, where respondents can directly answer ``Yes'', ``No'', or ``In process''. The ``In Process'' category for capturing meaningful intermediate states in administrative tasks is already underway. In contrast, a 5-point Likert scale, ranging from 1 (strongly disagree) to 5 (strongly agree), was used for items related to leader involvement, organizational values, operational stability, workplace quality, and economic performance.

The data indicate that most business owners or managers are female, accounting for 62.4 % of participants. This data illustrates that the majority of MSEs are managed by women, in line with the national MSE profile of 71.69 %. Regarding age, most owners are over 40 (55.8 %), followed by those aged 30 to 40 (31.1 %), and only 13.1 % are under 30. The food and beverage sector is the most dominant among the surveyed businesses, comprising 56.5 %, which aligns with national data showing it is the most dominant at 44.02 %. In comparison, wholesale and retail, fashion, and other services are less represented, at 26.6 % and 16.8 %, respectively. Furthermore, 74.5 % have been operational for over 3 years, while only 5.6 % have been operational for <1 year (see Table 3).

The data in this dataset is divided into two parts. The first part (Table 1) includes factor loadings and standard deviation for each item, and the total variance contributed by each factor. Table 1 indicates that the questionnaire data on organizational health comprises six factors: (1) organizational values, (2) leader involvement, (3) institutional resources, (4) operational stability, (5) work environment quality, and (6) economic performance. The first factor includes nine items, accounting for 13.04 % of the variance. The second factor consists of 6 items, contributing 10.62 % of the variance, and the third factor also has six items, explaining 10.34 % of the variance. The fourth factor contains 5 items, representing 8.52 % of the variance, while the fifth factor includes five items, accounting for 8.23 % of the variance. The sixth factor comprises four items, contributing 8.05 % of the variance. Collectively, these factors explain a total variance of 55.80 % and KMO Measure of Sampling Adequacy of 0.890.

**Table 1:** Factor loadings, variance explained, mean, and standard deviation.

No	Statements	Factor						Mean	SD
item		1	2	3	4	5	6		
Organizational values									
OH18	Feel free to talk about work difficulties	.356						2.890	1.398
OH19	Family atmosphere	.544						3.430	1.194
OH20	Morale	.709						3.550	1.201
OH21	Helping each other	.690						3.290	1.242
OH22	Mutual trust between employees	.772						3.630	1.284
OH23	Teamwork	.760						3.540	1.286
OH24	Respect each other	.677						3.430	1.233
OH25	Work is worship	.377						4.190	1.201
OH26	Working capital	.699						3.470	1.319
Leader involvement									
OH7	Personal closeness with employees		.587					2.850	.941
OH8	Role model		.781					2.950	.972
OH9	Open to employee ideas		.680					3.020	1.088
OH10	Directly involved		.732					2.930	1.092
OH11	Involved in training new employees		.720					2.900	1.081
OH12	Treats employees fairly		.779					3.050	1.031
Institutional resources									
OH1	Business Identification Number			.811				1.990	.647
OH2	Government funding assistance			.691				1.850	.634
OH3	Participation in government activities			.427				1.530	.702
OH4	Merchant cooperation			.750				1.950	.604
OH5	Using digital marketing			.829				1.840	.734
OH6	Source of financial			.723				1.590	.837
Operational stability									
OH27	Employee salaries				.785			2.430	.909
OH28	Equipment/machinery				.719			2.420	.890
OH29	Supply uncertainty				.704			2.370	.945
OH30	Demand uncertainties				.718			2.420	.941
OH31	Long term relationship with customers				.615			2.320	.937
The quality of the workplace									
OH13	Job-related accidents					.544		3.160	1.076
OH14	Working hours					.690		3.210	1.012
OH15	Workload					.771		3.400	1.034
OH16	Physical environment					.753		3.430	1.037
OH17	Absenteeism					.800		3.360	1.031
Economics performance									
OH32	Market reach						.657	1.790	.836
OH33	Sales growth						.853	1.830	.873
OH34	Liability						.832	1.700	.895
OH35	Cash Flow						.848	1.900	.846
Explained variance		13.040	10.620	10.340	8.520	8.230	7.950		
Eigenvalues		11.170	10.560	9.270	8.570	8.160	8.050		
KMO Measure of Sampling Adequacy		.890							
RSMEA= 0.049; TLI= 0.941									

The second part (Table 2) provides the label for each factor, the number of items per factor, mean, standard deviation, and reliability indices, including Cronbach's alpha, average variance extracted (AVE), and the Heterotrait-Monotrait (HTMT) ratio of correlations for each factor. The first factor, organizational values, ​​consists of nine items, with a mean of 3.41 and a standard deviation of 0.904, internal consistency (Cronbach's Alpha = 0.904), and AVE of 0.419. The second factor, leader Involvement, consists of six items, with a mean of 2.95, a standard deviation of 0.831, internal consistency Cronbach's Alpha = 0.889, and AVE = 0.582. The third factor, institutional resources, consists of six items, with a mean of 1.79, a standard deviation of 0.53, an internal reliability Cronbach's Alpha of 0.854, and an AVE of 0.524. Fourth, operational stability has five items: a mean of 2.39, a standard deviation of 0.757, Cronbach's Alpha = 0.877, and an AVE of 0.590. Fifth, the quality of the workplace environment factor includes six items, with a mean of 3.31, standard deviation of 0.845, Cronbach's Alpha = 0.873, and an AVE of 0.584. Lastly, economic performance includes four items: a mean of 1.81, standard deviation of 0.753, Cronbach's Alpha = 0.895, and AVE = 0.691.

**Table 2:** Reliability indices, AVE, and Heterotrait-monotrait (HTMT) ratio of correlations.

No	Factors	Number of items	Mean	SD	α	AVE	1	2	3	4	5	6
1	Organizational values	9	3.410	.904	.853	.419	1	.319	.154	.324	.264	.145
2	Leader involvement	6	2.950	.831	.889	.582	.319	1	.121	.486	.472	.283
3	Institutional resources	6	1.790	.530	.854	.524	.154	.121	1	.185	.281	.067
4	Operational stability	5	2.390	.757	.877	.590	.324	.486	.185	1	.380	.434
5	The quality of the workplace	5	3.310	.845	.873	.584	.264	.472	.281	.380	1	.129
6	Economic performance	4	1.810	.753	.895	.691	.145	.283	.067	.434	.129	1

**Table 3:** Characteristics of micro and small enterprises in this study.

	Sample (*n* = 428)		National Population (*N* = 4,4 million*)	
	Counts	Percent	Counts	Percent
*Gender of business owner/manager*				
Male	161	37.6	1245,640	28.31
Female	267	62.4	3154,360	71.69
*Age of business owner/manager*				
< 30 yrs	56	13.1	n.a	-
30 - 40 yrs	133	31.1	n.a	-
> 40 yrs	239	55.8	n.a	-
*Type of business*				
Food and Beverage	242	56.5	1936,880	44.02
Fashion	72	16.8	594,440	13.51
Wholesale and retail	114	26.6	n.a	-
*Company age*				
< 1 yr	24	5.6	n.a	-
1 - 3 yrs	85	19.9	n.a	-
> 3 yrs	319	74.5	n.a	-

Notes: * estimation based on the profile of Indonesian micro and small businesses in 2024 [14].

## 4 Experimental Design, Materials and Methods

The study used a survey design that collected data at different times and relied on convenience sampling. Although this method has its limitations, we chose the convenience sampling for three main reasons. First, this is a preliminary study aimed at exploring the basic structure of an organizational health measurement model before conducting larger, more detailed studies. Since this research is in its early stages, speed and flexibility are more important than generalizability [[8], [9], [10], [11]]. Second, time constraints necessitated that researchers gather data quickly to reduce the workload associated with more complex sampling methods [10,11]. Third, there is limited comprehensive data, especially in the micro sector, which often operates informally and is not fully registered in local government data in Indonesia. Convenience sampling enables researchers to collect relevant data despite these challenges by focusing on respondents who are easy to reach [8,10,12].

In the initial phase, our research team conducted a preliminary survey to secure permission from business owners in Jakarta and West Java, Indonesia. Participants were provided with a cover letter outlining the study’s objectives, data-handling procedures, anonymity guarantees, and clear instructions, and they voluntarily participated in the survey. Data were collected from two distinct sources—owners and employees—to mitigate common-method bias across two time points. Additionally, different rating scales were employed, tailored to the specific context of the question items [13].

At the first time point, micro and small enterprise (MSE) owners completed a paper-based questionnaire guided by the field team. From the collected responses, 458 MSE owners provided data on institutional resources, work environment quality, and operational stability. Subsequently, 1 to 2 employees from each business were randomly selected, and their contact information was collected to administer an online questionnaire later. Four hundred thirty-two employees responded to items measuring leader involvement, operational stability, and organizational values. This multi-source, multi-temporal approach effectively reduced potential biases associated with single-source data collection. After excluding cases with unmatched owner-employee pairs, the final dataset comprised 428 unique one-to-one matched sets of owner-employee ratings. These matched sets were utilized for the initial testing and validation of the organizational health instrument.

Exploratory factor analysis (EFA) was conducted to identify the questionnaire's underlying factor structure. Cronbach's Alpha was calculated to assess internal consistency, the Average Variance Extracted (AVE) was used to evaluate convergent validity, and the Heterotrait-Monotrait (HTMT) Ratio was applied to assess discriminant validity using JAMOVI software version 2.3.21.

## Limitations

This dataset has limitations due to its convenience sampling method in two Indonesian provinces, which affects the generalizability of the findings for all MSE contexts. As an initial exploration of organizational health (OH) in MSEs, the research uses Exploratory Factor Analysis to identify preliminary factor structures. Further validation through Confirmatory Factor Analysis on larger, more representative samples is needed to establish robust psychometric properties for the proposed OH model.

## Ethics Statement

The authors confirm that the work presented in this dataset is original and has not been submitted, either in whole or in part, for publication elsewhere. All listed authors have approved the manuscript for publication. Ethical approval was obtained from the ethics committee of Sekolah Tinggi Ilmu Ekonomi Indonesia Jakarta (Dr. Rimi Gusliana Mais). This study employed a self-report questionnaire to engage human participants, ensuring compliance with Indonesian national law and adherence to the ethical principles outlined in the Helsinki Declaration. Participation was voluntary, and participants were compensated with the equivalent of USD 2 for their time. Anonymity was strictly maintained throughout the data collection and analysis processes. All participants received a cover letter detailing the study’s objectives, procedures for handling their data, assurances of anonymity, and clear instructions for completing the questionnaire.

## Credit author statement

**Uun Sunarsih:** Data collection, Validation, Conceptualization, Methodology. **Hendryadi**: Methodology, Validation, Writing – original draft. **Irsan Tricahyadinata**: Supervision, Investigation, Reviewing and Editing. **Syahrul Effendi:** Data collection, Visualization. **Faris Faruqi:** Data collection, Software. **Harry Budiantoro**: Data collection, Investigation. **Santi Retno Sari**: Data collection, Investigation.

## Data Availability

Research DataTable 1, Table 2 (Original data).

## Appendix Supplementary materials

Image, application 1
