# Supplementary material for: Dataset on the exploratory factor structure of organizational health performance for micro and small enterprises: The initial research stage
Source: Data Brief. 2025 Nov 21;64:112317. doi: 10.1016/j.dib.2025.112317 (PMC12720095; doi:10.1016/j.dib.2025.112317)
Supplement: Supplementary file 1 [file mmc1.docx]

**RESEARCH INSTRUMENT**

**OWR OWNER/MANAGER**

*Institutional resources*

| No | Statements | No | In Process | Yes |
| --- | --- | --- | --- | --- |
| 1 | Does your business have a Business Identification Number (NIB)? |  |  |  |
| 2 | Have you received any government funding assistance to support your business? |  |  |  |
| 3 | Have you participated in any government-organized activities (e.g., training, workshops, or exhibitions)? |  |  |  |
| 4 | Do you collaborate with other merchants or businesses to grow your business? |  |  |  |
| 5 | Do you use digital marketing (e.g., social media, websites, or online ads) to promote your business? |  |  |  |
| 6 | What is the primary source of financial capital for your business? |  |  |  |

*Operational stability*

| No | Statements | **Strongly disagree** | **Disagree** | **Uncertain or neutral** | **Agree** | **Strongly agree** |
| --- | --- | --- | --- | --- | --- | --- |
| 1 | Working capital is adequate for daily business operations |  |  |  |  |  |
| 2 | The company is able to pay employee salaries on time |  |  |  |  |  |
| 3 | The equipment/machinery is in good condition and functions optimally |  |  |  |  |  |
| 4 | The business supply chain runs smoothly without significant disruptions |  |  |  |  |  |
| 5 | The demand for products/services is predictable. |  |  |  |  |  |
| 6 | The quality of business's long-term relationships with customers |  |  |  |  |  |
| 7 | Our business's ability to reach new markets or customers is excellent |  |  |  |  |  |

*Economics and financial performance*

| No | Statements | **Strongly disagree** | **Disagree** | **Uncertain or neutral** | **Agree** | **Strongly agree** |
| --- | --- | --- | --- | --- | --- | --- |
| 1 | Our business's ability to reach new markets or customers is excellent |  |  |  |  |  |
| 2 | Our business's sales growth over the past year has been very satisfactory |  |  |  |  |  |
| 3 | Our business's liabilities (e.g., loans, debts) are very manageable |  |  |  |  |  |
| 4 | Our business's cash flow situation is very healthy and stable |  |  |  |  |  |

***FOR EMPLOYEES***

Leader involvement

| No | Statements | **Strongly disagree** | **Disagree** | **Uncertain or neutral** | **Agree** | **Strongly agree** |
| --- | --- | --- | --- | --- | --- | --- |
| 1 | Your leader to employees on a personal level |  |  |  |  |  |
| 2 | Your leader act as a role model for employees |  |  |  |  |  |
| 3 | Your leader to implementing ideas or suggestions from employees |  |  |  |  |  |
| 4 | Your leader directly involved in day-to-day work |  |  |  |  |  |
| 5 | Your leader actively participate in training new employees |  |  |  |  |  |
| 6 | Your leader treat employees fairly when giving punishments and rewards |  |  |  |  |  |

Workplace environment quality

| No | Statements | **Strongly disagree** | **Disagree** | **Uncertain or neutral** | **Agree** | **Strongly agree** |
| --- | --- | --- | --- | --- | --- | --- |
| 1 | Workplace is free from job-related accidents |  |  |  |  |  |
| 2 | I am satisfied with the working hours set in this company |  |  |  |  |  |
| 3 | My workload is manageable |  |  |  |  |  |
| 4 | The physical working conditions (e.g., noise, lighting, temperature) are comfortable |  |  |  |  |  |
| 5 | Most of employees are responsible and rarely miss work without valid reasons |  |  |  |  |  |
| 6 | Your leader treat employees fairly when giving punishments and rewards |  |  |  |  |  |

Organizational values

| No | Statements | **Strongly disagree** | **Disagree** | **Uncertain or neutral** | **Agree** | **Strongly agree** |
| --- | --- | --- | --- | --- | --- | --- |
| 1 | I feel free to talk about work-related difficulties with my leader or colleagues |  |  |  |  |  |
| 2 | The atmosphere in my workplace is positive and supportive |  |  |  |  |  |
| 3 | The morale of employees in my workplace is generally high |  |  |  |  |  |
| 4 | Employees in my workplace help each other when needed |  |  |  |  |  |
| 5 | The level of trust among employees in my workplace is very strong |  |  |  |  |  |
| 6 | Teamwork in my workplace is effective |  |  |  |  |  |
| 7 | Employees in my workplace treat each other with respect |  |  |  |  |  |
| 8 | Employees in my workplace view their work as a form of dedication or worship |  |  |  |  |  |
